# Cardiometabolic risk factors predict cerebrovascular health in older adults: results from the *Brain in Motion* study

**DOI:** 10.14814/phy2.12733

**Published:** 2016-04-25

**Authors:** Amanda V. Tyndall, Laurie Argourd, Tolulope T. Sajobi, Margie H. Davenport, Scott C. Forbes, Stephanie J. Gill, Jillian S. Parboosingh, Todd J. Anderson, Ben J. Wilson, Eric E. Smith, David B. Hogan, Michael D. Hill, Marc J. Poulin

**Affiliations:** ^1^ Department of Physiology & PharmacologyCumming School of MedicineUniversity of CalgaryCalgaryAlbertaT2N 4N1Canada; ^2^ Hotchkiss Brain InstituteCumming School of MedicineUniversity of CalgaryCalgaryAlbertaT2N 4N1Canada; ^3^ Department of Clinical NeurosciencesCumming School of MedicineUniversity of CalgaryCalgaryAlbertaT2N 4N1Canada; ^4^ Department of Community Health SciencesCumming School of MedicineUniversity of CalgaryCalgaryAlbertaT2N 4Z6Canada; ^5^ Department of Medical GeneticsCumming School of MedicineUniversity of CalgaryCalgaryAlbertaT2N 4N1Canada; ^6^ Alberta Children's Hospital Research Institute for Child and Maternal HealthUniversity of CalgaryCalgaryAlbertaT3B 6A8Canada; ^7^ Department of Cardiac SciencesCumming School of MedicineUniversity of CalgaryCalgaryAlbertaT2N 4N1Canada; ^8^ Libin Cardiovascular Institute of AlbertaCumming School of MedicineUniversity of CalgaryCalgaryAlbertaT2N 4N1Canada; ^9^ Department of MedicineCumming School of MedicineUniversity of CalgaryCalgaryAlbertaT2N 4N1Canada; ^10^ Faculty of KinesiologyUniversity of CalgaryCalgaryAlbertaT2N 1N4Canada

**Keywords:** Aging, brain health, cerebrovascular regulation, metabolic syndrome

## Abstract

Aging and physical inactivity are associated with an increased risk of developing metabolic syndrome (MetS). With the rising prevalence of MetS, it is important to determine the extent to which it affects cerebrovascular health. The primary purpose of this report is to examine the impact of MetS on cerebrovascular health (resting cerebral blood flow (CBF) peak velocity (${\bar{V}}_{P}$), cerebrovascular conductance (CVC), and CBF responses to hypercapnia) in healthy older adults with normal cognition. A secondary goal was to examine the influence of apolipoprotein E (*APOE*) *ε*4 expression on these indices. In a sample of 258 healthy men and women older than 53 years, 29.1% met criteria for MetS. MetS, sex, and age were found to be significant predictors of CVC, and ${\bar{V}}_{P}$, MetS, and *APOE* status were significant predictors of ${\bar{V}}_{P}$‐reactivity, and CVC‐reactivity was best predicted by MetS status. After controlling for these factors, participants with MetS demonstrated lower cerebrovascular measures (CVC, ${\bar{V}}_{P}$, CVC‐reactivity, and ${\bar{V}}_{P}$‐reactivity) compared to participants without MetS. *APOE ε*4 carriers had higher ${\bar{V}}_{P}$‐reactivity than noncarriers. These results provide evidence that cardiometabolic and vascular risk factors clustered together as the MetS predict measures of cerebrovascular health indices in older adults. Higher ${\bar{V}}_{P}$‐reactivity in *APOE ε*4 carriers suggests vascular compensation for deleterious effects of this known risk allele for Alzheimer's disease and stroke.

## Introduction

A number of risk factors for cardiovascular disease (CVD), stroke, and type 2 diabetes mellitus (*e.g*., hypertension, dyslipidemia, hyperglycemia, and abdominal obesity) when clustered together are called metabolic syndrome (MetS) (Alberti et al. 2009). In the Canadian Health Measures Survey, 21% of Canadians between the ages of 18 and 79 years were classified as having MetS (Statistics Canada, 2014), with an even higher prevalence (34%) reported in the USA (Ervin 2009). Prevalence increases with age, a sedentary lifestyle, and obesity (Rao et al. 2014). Lifestyle factors other than sedentary or physically inactive behavior, such as alcohol consumption and tobacco use have also been shown to affect the development of MetS (Slagter et al. 2014) as does sociodemographic factors such as education (Rao et al. 2014). The prevalence of MetS is rising worldwide in parallel with population aging (Alberti et al. 2009; Kaur 2014).

Aging is associated with declines in resting cerebral blood flow (CBF) (Chen et al. 2011; Zimmerman et al. 2014) and cerebrovascular reactivity to hypercapnia (Barnes et al. 2013) that are associated with an increased risk of subsequent cognitive decline and dementia (Lautenschlager et al. 2012), stroke (Gupta et al. 2012), and cardiovascular and all‐cause mortality (Portegies et al. 2014). MetS may accelerate these adverse age‐related changes in cerebrovascular health (Giannopoulos et al. 2010; Farooqui et al. 2012; Birdsill et al. 2013). Birdsill et al. (2013) found a 15% reduction in mean gray matter CBF in middle aged to older adults (mean age = 60.4 years) with MetS, while Giannopoulos et al. (2010) demonstrated impairment in vasomotor reactivity to hypercapnia in atherosclerotic patients with MetS. These results provide potential mechanisms for the relationship between MetS and a steeper trajectory of cognitive decline beyond normal cognitive aging (Yates et al. 2012).

There is growing interest in the association between genetic risk and cerebrovascular aging. Large genome‐wide association studies have revealed that many of the identified candidate genes associated with the risk of Alzheimer disease (AD) encode proteins that are involved in lipid metabolism (Wollmer 2010) that may have downstream consequences on the vascular system through mechanisms such as oxidative stress or atherosclerosis (Farooqui et al. 2012; Teixeira et al. 2014). Specifically, apolipoprotein E (APOE), the most ubiquitous cholesterol transport protein in the central nervous system (Wollmer 2010) has been identified as the most important susceptibility gene for late‐onset AD risk (Yu et al. 2014). Along with increased susceptibility to AD, carriers of the *APOE ε*4 polymorphism are also at higher risk of developing CVD (Dore et al. 2009). Recent studies have provided evidence that *APOE ε*4 status modifies the interaction of type 2 diabetes and/or prediabetic states with cognitive function (Dore et al. 2009). These results suggest an interaction between modifiable risk factors such as cardiometabolic characteristics and genetic factors on brain health outcomes. Since MetS is related to a disruption in triglyceride‐rich lipoproteins and high‐density lipoprotein, the *APOE ε*4 polymorphism may play an important modifying role in the relationship between MetS and cerebrovascular health. The *APOE ε*4 polymorphism has been demonstrated to be a risk factor for MetS (Sima et al. 2007) with higher CVD prevalence among those already with MetS and carrying the allele (Teixeira et al. 2014). Studies examining the interactive effects of CBF and *APOE ε*4 genotype have observed regional increases in CBF in older adults with (Wierenga et al. 2012) and without (Zlatar et al. 2014) mild cognitive impairment (MCI). However, little is known about the interaction of cardiometabolic risk factors (i.e., MetS) and AD and CVD genetic risk factors (i.e., *APOE ε*4 polymorphism) on cerebrovascular indices. The effects of MetS on cerebrovascular function and the influence of *APOE* genotype in healthy older adults without clinically overt vascular disease or cognitive impairments are not well understood.

The primary purpose of this article is to report on the impact of MetS on indices of cerebrovascular health (e.g., CBF peak velocity [${\bar{V}}_{P}$] and cerebrovascular conductance [CVC], and CBF responses to hypercapnia) in older adults. The secondary purpose was to determine the extent to which *APOE ε*4 genotype affects the relationship between MetS and cerebrovascular health. While other genes have been identified to contribute to MetS (Joy et al. 2008), we were interested in examining *APOE ε*4 because of its association with increased risk for late‐onset AD, CVD, and cerebrovascular disease. We hypothesized that individuals with MetS would have impaired vascular regulation as reflected by lower resting ${\bar{V}}_{P}$ and CVC as well as lower ${\bar{V}}_{P}$ and CVC responses to hypercapnia compared to participants without MetS. Further, we hypothesized that carriers of the *APOE ε*4 genotype would have increased CBF as compared to noncarriers, possibly related to compensatory mechanisms prior to the onset of cognitive decline (Luckhaus et al. 2008; Dai et al. 2009; Wierenga et al. 2012). Exploratory analyses were also conducted to examine the impact of hyperinsulinemia and insulin resistance (homeostatic model of assessment: HOMA), which are associated with the MetS, on measures of cerebrovascular health (Ryan et al. 2013).

## Research Design And Methods

Cross‐sectional observational data collected as part of a larger prospective cohort study examining the impact of exercise training on cerebrovascular health and cognitive function in healthy middle‐aged and older adults (mean = 65.9 years, SD = 6.5 years; *Brain in Motion* [BIM] Study Tyndall et al. 2013) were used for this study. All data reported in this study were collected prior to the exercise intervention phase of the BIM study (i.e., “baseline” phase of testing).

### Ethical approval

Data from 263 men and postmenopausal women aged 54–93 years who volunteered for the BIM study were included in this report. All participants provided written informed consent. This study conforms to the standards set by the *Declaration of Helsinki*. The study protocol was approved by the University of Calgary Conjoint Health Research Ethics Board.

### Inclusion/Exclusion criteria

For inclusion into the BIM study, potential participants had to meet the following criteria: (1) age >50 years; (2) reported <30 min of moderate exercise 4 days per week or 20 continuous minutes of vigorous exercise 2 days per week; (3) able to walk independently outside and up and down at least 20 stairs; (4) not diagnosed with clinically evident cardiovascular/cerebrovascular disease(s), asthma and/or type I diabetes mellitus; (5) score ≥ 24 on the Montreal Cognitive Assessment (Nasreddine et al. 2005); (6) nonsmoker for at least 12 months; (7) no major surgery or trauma in the last 6 months; (8) free of neurological disorders such as multiple sclerosis; and (9) clearance obtained from their attending health care professional to participate in the study. Prior to study entry, each participant was assessed by a study physician and their medications recorded.

### Demographic and behavioral variables

A description of participants’ education level was comprised of three variables: number of years of education, highest degree/diploma obtained, and main occupation. A binary variable was created based on the International Standard Classification of Education of 1997 (ISCED‐1997), in which the participants were grouped based on having; (1) a high school level or equivalent degree; and (2) a Tertiary or higher level of education.

Alcohol consumption was reported in the diet history questionnaire (DHQ), a self‐administered food frequency questionnaire (Csizmadi et al. 2007), from which alcohol consumption (g/day) was established. According to the National Institute of Alcohol Abuse and Alcoholism, one standard drink is equivalent to 14 g of alcohol (National Institute on Alcohol Aduse and Alcoholism 2015). For the analysis, we considered a light drinker as reporting ≤2.2 drinks per day and a drinker as reporting more drinks per day (Abel et al. 1998). Tobacco use (smoking history) was assessed during participants’ phone interview for eligibility for the BIM study, in which they were classified into two categories – ever smoked or never smoked in their lifetime.

### Metabolic syndrome

The presence of MetS was defined according to the joint statement of the International Diabetes Federation Task Force on Epidemiology and Prevention, National Heart, Lung, and Blood Institute; American Heart Association, World Heart Federation, International Atherosclerosis Society, and International Association for the Study of Obesity (Alberti et al. 2009). Specifically, MetS was defined as having ≥3 of the following clinical features: (1) serum triglycerides ≥1.69 mmol/L; (2) fasting serum glucose ≥5.6 mmol/L or on an antihyperglycaemic medication; (3) waist circumference ≥102 cm in men or ≥88 cm in women; (4) BP ≥ 130/85 mmHg or on an antihypertensive medication; and/or (5) serum high‐density lipoprotein (HDL) cholesterol <1.04 mmol/L in men or <1.29 mmol/L in women or on a lipid lowering medication (Alberti et al. 2009). Following a 12‐h fast, venous blood samples were collected from the antecubital vein, centrifuged, aliquoted, and stored at −80°C for later analysis. Fasting serum glucose, serum triglycerides, serum insulin levels, high‐sensitivity C‐reactive protein (hsCRP), total cholesterol, low‐density lipoprotein (LDL) cholesterol, and high‐density lipoprotein (HDL) cholesterol were determined by Calgary Laboratory Services using standardized laboratory procedures. Insulin resistance was quantified using the homeostatic model assessment (HOMA) (Matthews et al. 1985), which is derived from fasting glucose and insulin.

### Four indices of cerebrovascular function are the primary outcomes

The primary outcomes of this study were four indices of cerebrovascular health: peak systolic velocity in the middle cerebral artery (${\bar{V}}_{P}$), cerebrovascular conductance (CVC), ${\bar{V}}_{P}$‐reactivity, and CVC‐reactivity. CVC is the change in ${\bar{V}}_{P}$ divided by the change in mean arterial blood pressure reported from the +1 mmHg stage. ${\bar{V}}_{P}$‐reactivity is the absolute change in ${\bar{V}}_{P}$ divided by the absolute change in measured $P_{{\mathrm{ETCO}}_{2}}$ from +1 mmHg to +8 mmHg. CVC‐reactivity is the absolute change in CVC divided by the absolute change in measured $P_{{\mathrm{ETCO}}_{2}}$ from +1 mmHg to +8 mmHg. Both CVC and CVC‐reactivity account for variation in blood pressure between participants. A higher value in all the cerebrovascular measures (${\bar{V}}_{P}$, CVC, ${\bar{V}}_{P}$‐reactivity, CVC‐reactivity) is considered superior for cerebrovascular health (Davenport et al. 2012).

Measurements were taken prior to the determination of MetS status to ensure that the tester was blinded on this. Participants refrained from eating or drinking anything other than water for two hours prior to testing and from engaging in exercise on the day of testing. Participants were seated in a semireclined position after a 10‐min rest period during data collection. Blood flow velocity (${\bar{V}}_{P}$) through the middle cerebral artery (MCA) was measured noninvasively using a 2‐MHz transcranial Doppler ultrasound probe (TCD; Toc Neurovision^™^, Multigon Industries, INC., Yonkers, NY). The MCA was located by placing the TCD probe over the temporal region superior to the zygomatic process in close proximity to the ear, using techniques previously described (Aaslid et al. 1982). An index of cerebral blood flow was obtained from the peak middle cerebral blood flow velocity (${\bar{V}}_{P}$) (Brown et al. 2010). A 3‐lead ECG (Micromon 7142 B, Kontron Medical, Milton Keynes, UK), a beat‐by‐beat finger‐pulse photoplethysmography finometer (Finapres Medical Systems, Amsterdam, The Netherlands), and a finger pulse oximeter (3900p, Datex‐Ohmeda, Madison, WI) were used to continuously monitor heart rate, blood pressure, and arterial hemoglobin saturation, respectively. An automated blood pressure was taken at the contralateral brachial artery for calibration of the beat‐by‐beat measures.

Baseline end‐tidal respiratory measures ($P_{{\mathrm{CO}}_{2}}$ and $P_{O_{2}}$) were recorded (Chamber, University Laboratory of Physiology, Oxford, UK), during a 10‐min air breathing period, and were used to determine the desired end‐tidal partial pressure of carbon dioxide ($P_{{\mathrm{ETCO}}_{2}}$) and oxygen ($P_{{\mathrm{ETO}}_{2}}$) for determination of cerebrovascular responses during a euoxic hypercapnia test. With their nose occluded, each participant breathed room air through a mouthpiece connected to a dynamic end‐tidal forcing (DEF) system. A fine capillary connected to a mass spectrometer (AMIS 2000, Innovision, Odense, Denmark) was inserted into the mouthpiece to continuously assess the fraction of CO_2_ and O_2_ within the respired gases.

The euoxic hypercapnia test comprised of two 3‐min step increases in carbon dioxide. For the first minute, participants breathed room air only. This time period was followed by a 5‐minute baseline period during which $P_{{\mathrm{ETCO}}_{2}}$ was held at +1 mmHg above participants’ resting, air breathing $P_{{\mathrm{ETCO}}_{2}}$ value. Following baseline, $P_{{\mathrm{ETCO}}_{2}}$ was increased to +5 mmHg above air breathing values for three minutes and then was increased to +8 mmHg for another three minutes. $P_{{\mathrm{ETO}}_{2}}$ was maintained at baseline levels throughout. Precise control of desired $P_{{\mathrm{ETCO}}_{2}}$ and $P_{{\mathrm{ETO}}_{2}}$ values was achieved continuously via the DEF system using sophisticated software (BreatheM v2.40, University of Laboratory of Physiology, Oxford, UK) as previously described (Poulin et al. 1996).

### Maximal aerobic capacity, anthropometric measurements, and apolipoprotein E genotype are explanatory variables

Maximal aerobic capacity ($\dot{V}O_{2\max}$) was assessed to determine participants’ physical fitness. The BIM study aims to recruit sedentary older adults who may be at risk for the development of cardiovascular disease and type II diabetes and would strongly benefit from a structured aerobic exercise intervention. $\dot{V}O_{2\max}$was assessed using a modified Bruce protocol (Paterson et al. 1999; Tyndall et al. 2013) on a motorized treadmill; this assessment was completed during a separate laboratory visit. Attainment of $\dot{V}O_{2\max}$ was indicated by a plateau in oxygen uptake with increasing work rate (<2 mL/kg/min), a respiratory exchange ratio (RER) of at least 1.15, age‐predicted maximal heart rate (210 − (age*0.65)), and volitional exhaustion (Tyndall et al. 2013). During the test, expired air was analyzed for O_2_ and CO_2_ using a calibrated metabolic system (ParvoMed True Max 2400, Utah). Anthropometric measurements measured at baseline using standardized methods included height, mass, body mass index (BMI; mass (kg)/height (m)^2^), waist circumference (Brubaker et al. 2006), and percentage body fat (bioelectrical impedance analysis).

Genotyping was conducted on 242 samples; 10 participants did not consent to optional genetic analysis. Genomic DNA was extracted from buffy coats obtained from whole blood using standard protocols (Qiagen Gentra Puregene Blood Kit). Sequence data was generated by PCR‐amplification followed by Sanger sequencing (BigDye v1.1 Cycle Sequencing Kit Applied Biosystems) on ABI 3130XLGenetic Analyzer (Applied Biosystems). Detailed protocols are available upon request. Mutation Surveyor DNA Variant Analysis software (SoftGenetics, LLC, State Collage, PA) was used to identify variants within the sequence. *APOE ε*2, *ε*3, and *ε*4 alleles were established by manually combining the alleles from the single‐nucleotide polymorphisms, NM_000041.2:c.388T>C (p.Cys130Arg; rs429358) and c.526C>T (p.Arg176Cys; rs7414) as follows: at nucleotides 388 and 526 (amino acids 130 and 176), *ε*2 = TT (CysCys), *ε*3 = TC (CysArg), and *ε*4 = CC (ArgArg). For this study, we were interested in examining the effects of carrying an *APOE ε*4 allele on cerebrovascular function. Participants were grouped into *APOE ε*4^+^ included *ε*3/*ε*4 and *ε*4/*ε*4 genotypes, while *APOE ε*4^‐^ included *ε*2/*ε*2, *ε*2/*ε*3, and *ε*3/*ε*3 genotypes. Due to the opposite effects of the *ε*2 and *ε*4 alleles, participants with *ε*2/*ε*4 genotype were removed from the sample (*n* = 6) (Corder et al. 1994).

### Statistical analyses

**Figure 1:**
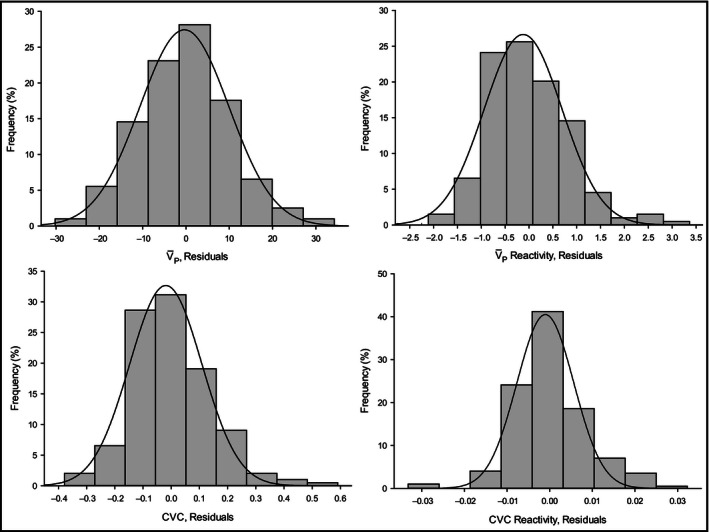
Normality assessment of the cerebrovascular health indices. Histograms display the distribution (frequency, %) of each of the cerebrovascular measure residuals compared to the Normal distribution represented by a solid line. CVC, cerebrovascular conductance (cm/s/mmHg); ${\bar{V}}_{P}$, resting cerebral blood flow peak velocity (cm/s); ${\bar{V}}_{P}$‐reactivity (cm/s/mmHg), cerebral blood flow reactivity to hypercapnic challenge from +1 torr to +8 torr; CVC‐reactivity (cm/s/mmHg/mmHg), cerebrovascular conductance reactivity to a hypercapnic challenge from +1 torr to +8 torr.

Means and standard deviations were used to summarize the continuous variables, while frequency distribution was used to summarize the categorical variables. Independent sample *t* tests were used to identify sex‐adjusted differences between MetS and the non‐MetS groups on continuous variables, while chi square test or Fisher's exact test was used to test between group differences on categorical variables. Multiple linear regression analysis was used to model the association between each cerebrovascular outcome and MetS after controlling for sex, age, education, smoking history, drinking status, and *APOE* genotype, which are factors associated with cerebrovascular indices as well as MetS (Strandgaard 1993; Brown et al. 2010; Gibson 2013). For each cerebrovascular health outcome, we assessed the tenability of the normal distributional assumption in the linear regression using visual inspection of the Q–Q plot of the residuals distribution (Fig. 1). White (1980) test was used to assess the tenability of the assumption of homoscedasticity (i.e., constant variance) for each linear regression. Conditional variance indices and variance inflation factor (Belsley et al. 1980) were used to assess the presence of multicollinearity among our potential predictors of cerebrovascular outcomes. Predictors with high conditional variance indices and VIF were excluded from the final model. The coefficient of determination (i.e., model *R* ^2^) was used to assess the proportion of variance in each cerebrovascular outcomes that is explained by the model predictors. Statistical significance of each predictor was evaluated at *α *= 0.05. All statistical analyses were conducted in SAS 9.3 (SAS Institute Inc, 2011).

## Results

Four participants had incomplete data due to difficulties insonating the middle cerebral artery and one participant was excluded for type I diabetes mellitus. These five participants were excluded from all analyses. Results are presented on the remaining 258 (*n* = 122 men; *n* = 136 women) participants.

**Figure 2:**
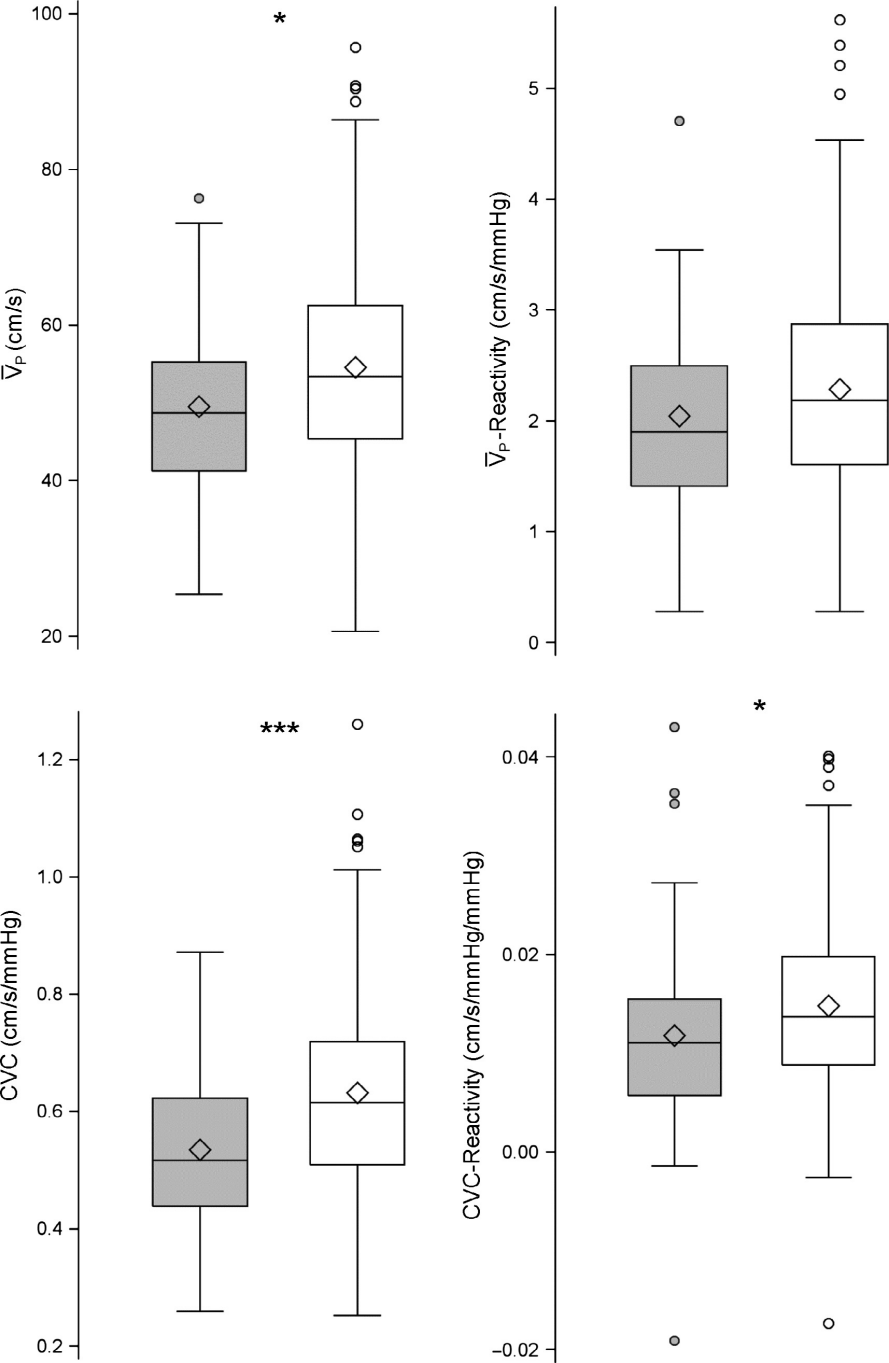
Comparison of the MetS (gray box; *n* = 75) versus No MetS (open box; *n* = 183) for the different vascular measurements, corrected for sex. Box plots display the first and third quartiles and whiskers represent the interquartile range. The mean is represented by the diamond within the box and the central horizontal line within the box represents the median of the data. Filled in circles (MetS) or open circles (No MetS) represent data that fall out of the interquartile range. *P <* *0.05, **P <* *0.01, ***P <* *0.001. CVC, cerebrovascular conductance (cm/s/mmHg); ${\bar{V}}_{P}$, resting cerebral blood flow peak velocity (cm/s); ${\bar{V}}_{P}$‐reactivity (cm/s/mmHg), cerebral blood flow reactivity to hypercapnic challenge from +1 torr to +8 torr; CVC‐reactivity (cm/s/mmHg/mmHg), cerebrovascular conductance reactivity to a hypercapnic challenge from +1 torr to +8 torr.

A description of participants’ characteristics by the presence of the MetS is presented in Table 1 and stratified by sex in Tables 2 and 3. Seventy‐five participants (29.1% of the total) met criteria for MetS. After correcting for sex, there were differences in drinking status, $\dot{V}O_{2\max}$, anthropometric, and biochemical measures between participants with and without MetS (Table 1). Univariate tests of differences in cerebrovascular health outcomes between Mets and No Mets revealed that individuals in the No MetS group had significantly higher mean ${\bar{V}}_{P}$, CVC, and CVC‐reactivity values than the MetS groups. But there were no significant differences in the mean ${\bar{V}}_{P}$‐reactivity values for both groups (Fig. 2). Variance inflation factors (VIF) and conditional indices were used to assess the potential for collinear among our model predictors. Using Belsley et al. (1980) criterion, predictors with VIF and conditional index greater than 10 were flagged for collinearity and removed from the multiple linear regressions. Our final regression model for each cerebrovascular outcomes included MetS, sex, age, education, smoking history, drinking status, and *APOE* genotype as model predictors (Table 4).

**Table 1:** Participant characteristics, metabolic profiles, blood pressures, and medications between participants without metabolic syndrome (No MetS) and those with metabolic syndrome (MetS)

Variables	No Mets (*n* = 183)		Mets (*n* = 75)		*P* value^a^
	*N*		*N*		
Participant characteristics					
Sex, No. (%)					
Male	77 (63.1)		45 (36.9)		0.009
Female	106 (77.9)		30 (22.06)		
Age, Mean (SD), years	183	65.8 (6.8)	75	66.0 (5.7)	0.84
[55–65], No. (%)	91 (49.7)		35 (46.7)		0.03
66 and +, No. (%)	92 (50.3)		40 (53.3)		
Weight, Mean (SD), kg	183	73.9 (13.6)	75	85.7 (13.4)	<0.001
BMI, Mean (SD), kg/m²	183	26.1 (3.5)	75	29.2 (3.4)	<0.001
Body fat, Mean (SD), %	177	31.4 (7.8)	75	33.4 (6.2)	<0.001
Waist circumference, Mean (SD), cm	181	91.8 (13.7)	75	104.0 (9.9)	<0.001
$\dot{V}O_{2\max}$, Mean (SD), mL/kg/min	182	26.7 (5.8)	74	24.7 (4.5)	<0.001
Education, No. (%)					
High school or equivalent	39 (21.3)		20 (26.7)		0.983^c^
Tertiary education	144 (78.7)		55 (73.3)		
Working status, No. (%)					
Working	63 (34.4)		26 (34.7)		0.82
Semi	16 (8.7)		8 (10.7)		
Retired	104 (56.8)		41 (54.7)		
Drinking status, No. (%)					
Light drinker	141 (95.3)		53 (81.5)		0.01
Drinker	7 (4.7)		12 (18.5)		
Smoking history, No. (%)					
Never smoked	105 (58.0)		34 (45.3)		0.21
Ever smoked	76 (42.0)		41 (54.7)		
Genetic characteristics					
*APOE*, No. (%)					
*ε*4^+^	45 (25.9)		18 (26.5)		0.98
*ε*4^−^	129 (74.1)		50 (73.5)		
Metabolic characteristics, Mean (SD)					
Glucose, mmol/L	183	5.2 (0.5)	74	6.0 (0.9)	<0.001
Triglycerides, mmol/L	183	1.1 (0.3)	75	1.9 (0.7)	<0.001
Cholesterol, mmol/L	183	5.4 (0.9)	75	5.2 (1.1)	0.68
HDL, mmol/L	183	1.8 (0.5)	75	1.3 (0.3)	<0.001
LDL, mmol/L	183	3.1 (0.7)	75	3.1 (1.0)	0.84
Total cholesterol/HDL	183	3.2 (0.8)	75	4.3 (1.3)	<0.001
Insulin, pmol/L	181	44.7 (23.7)	75	72.4 (36.6)	<0.001
HOMA	181	1.5 (0.9)	74	2.9 (1.7)	<0.001
hsCRP, mG/L	182	1.3 (1.6)	74	2.4 (3.4)	0.001
Blood pressure, Mean (SD), mmHg					
Systolic BP	183	122.0 (14.7)	75	132.0 (15.8)	<0.001
Diastolic BP	183	70.7 (8.3)	75	75.4 (8.5)	0.001
MAP	183	87.8 (9.4)	75	94.3 (9.6)	<0.001
Metabolic syndrome factors, No. (%)					
High waist circumference	70 (38.3)		61 (81.3)		<0.001
High triglycerides	7 (3.8)		47 (62.7)		<0.001
Low HDL	19 (10.4)		43 (57.3)		<0.001
Hypertensive	66 (36.1)		62 (82.7)		<0.001
High glucose	43 (23.5		59 (78.7)		<0.001
Medications, No. (%)					
Antihypertensive	30 (16.4)		39 (52.0)		<0.001
Antihyperglycemic^b^	1 (0.5)		7 (9.3)		<0.001
Antihyperlipidemic	11 (6.0)		19 (25.3)		<0.001

MetS, metabolic syndrome; BMI, Body mass index; $\dot{V}O_{2\max}$, Maximal oxygen uptake; *APOE*, Apolipoprotein E; HDL, high‐density lipoprotein cholesterol; LDL, Low‐density lipoprotein cholesterol; HOMA, Homeostatic model assessment; HsCRP, High‐sensitivity C‐reactive protein; BP, Blood Pressure; MAP, Mean arterial blood pressure.

a Calculated from *t* test or *χ* ^*2*^

b Probability calculated from *fisher exact* test.

c Interaction between MetS status and sex, *P* = 0.0091

**Table 2:** Participant characteristics of women without metabolic syndrome (No MetS) and those with metabolic syndrome (MetS)

Variables	No Mets (*n* = 106)		Mets (*n* = 30)		*P* value^a^
	*N*		*N*		
Participant characteristics					
Age, Mean (SD), years	106	65.5 (6.2)	30	64.7 (5.8)	0.94
[55–65], No. (%)	49 (46.2)		18 (60.0)		0.18
66 and +, No. (%)	57 (53.8)		12 (40.0)		
Weight, Mean (SD), kg	106	67.8 (11.4)	30	75.0 (11.0)	0.01
BMI, Mean (SD), kg/m²	106	25.7 (3.9)	30	28.4 (3.9)	0.001
Body fat, Mean (SD), %	102	36.5 (5.3)	30	39.2 (3.8)	0.041
Waist circumference, Mean (SD), cm	105	88.7 (12.3)	30	99.9 (9.7)	<0.001
$\dot{V}O_{2\max}$, Mean (SD), ml/kg/min	105	24.0 (4.8)	30	22.2 (3.1)	0.23
Education, No. (%)^b^					
High school or equivalent	25 (23.6)		3 (10.0)		0.10
Tertiary education	81 (76.4)		27 (90.0)		
Working status, No. (%)					
Working	33 (31.1)		8 (26.7)		0.87
Semi	8 (7.5)		2 (6.7)		
Retired	65 (61.3)		20 (66.7)		
Drinking status, No. (%)^b^					
Light drinker	85 (95.5)		22 (88.0)		0.14
Drinker	4 (4.5)		3 (12.0)		
Smoking history, No. (%)					
Never smoked	65 (62.5)		19 (63.3)		0.93
Ever smoked	39 (37.5)		11 (36.7)		
Genetic characteristics					
*APOE*, No. (%)					
Ɛ4^+^	23 (22.6)		7 (26.9)		0.64
Ɛ4^−^	79 (77.5)		19 (73.1)		
Metabolic characteristics, Mean (SD)					
Glucose, mmol/L	104	5.1 (0.4)	29	6.0 (1.0)	<0.001
Triglycerides, mmol/L	104	1.0 (0.3)	30	1.8 (0.6)	<0.001
Cholesterol, mmol/L	104	5.6 (0.8)	30	5.4 (1.1)	0.72
HDL, mmol/L	104	1.9 (0.5)	30	1.3 (0.3)	<0.001
LDL, mmol/L	104	3.2 (0.7)	30	3.3 (1.0)	0.95
Total cholesterol/HDL	104	3.0 (0.8)	30	4.3 (1.3)	<0.001
Insulin, pmol/L	103	43.4 (17.3)	30	70.9 (40.7)	<0.001
HOMA	103	1.4 (0.6)	29	2.8 (1.7)	<0.001
hsCRP, mG/L	104	1.5 (1.6)	30	2.5 (3.4)	0.12
Blood pressure, Mean (SD), mmHg					
Systolic BP	106	121.0 (16.2)	30	131.0 (14.9)	0.009
Diastolic BP	106	68.7 (8.3)	30	73.0 (9.8)	0.047
MAP	106	86.2 (9.9)	30	92.4 (10.1)	0.008
Metabolic syndrome factors, No. (%)					
High waist circumference	50 (47.2)		28 (93.3)		<0.001
High triglycerides^b^	3 (2.8)		16 (53.3)		<0.001
Low HDL	12 (11.3)		18 (60.0)		<0.001
Hypertensive	37 (34.9)		23 (76.7)		<0.001
High glucose	15 (14.2)		22 (73.3)		<0.001
Medications, No. (%)					
Antihypertensive	16 (15.1)		15 (50.0)		<0.001
Antihyperglycemic^b^	0 (0.0)		5 (16.7)		<0.001
Antihyperlipidemic^b^	4 (3.8)		5 (16.7)		0.021

MetS, metabolic syndrome; BMI, Body mass index; $\dot{V}O_{2\max}$, Maximal oxygen uptake; *APOE*, Apolipoprotein E; HDL, high‐density lipoprotein cholesterol; LDL, Low‐density lipoprotein cholesterol; HOMA, Homeostatic model assessment; HsCRP, High‐sensitivity C‐reactive protein; BP, Blood Pressure; MAP, Mean arterial blood pressure.

a Calculated from *t* test or *χ* ^*2*^

b Probability calculated from *fisher exact* test.

**Table 3:** Participant characteristics of men without metabolic syndrome (No MetS) and those with metabolic syndrome (MetS)

Variables	No Mets (*n* = 77)		Mets (*n* = 45)		*P* value^a^
	*N*		*N*		
Participant characteristics					
Age, Mean (SD), years	77	66.4 (7.6)	45	66.8 (5.5)	.99
[55–65], No. (%)	42 (54.6)		17 (37.8)		.074
66 and +, No. (%)	35 (45.5)		28 (62.2)		
Weight, Mean (SD), kg	77	82.2 (11.9)	45	92.9 (9.5)	<.001
BMI, Mean (SD), kg/m²	77	26.7 (2.8)	45	29.7 (2.9)	<.001
Body fat, Mean (SD), %	75	24.6 (5.0)	45	29.5 (4.0)	<.001
Waist circumference, Mean (SD), cm	76	96.3 (14.4)	45	107.0 (9.0)	<.001
$\dot{V}O_{2\max}$, Mean (SD), mL/kg/min	77	30.3 (5.0)	44	26.3 (4.6)	<.001
Education, No. (%)					
High school or equivalent	14 (18.2)		17 (37.8)		.016
Tertiary education	63 (81.8)		28 (62.2)		
Working status, No. (%)					
Working	30 (39.0)		18 (40.0)		.85
Semi	8 (10.4)		6 (13.3)		
Retired	39 (50.6)		21 (46.7)		
Drinking status, No. (%)^b^					
Light drinker	56 (94.9)		31 (77.5)		.01
Drinker	3 (5.1)		9 (22.5)		
Smoking history, No. (%)					
Never smoked	40 (51.9)		15 (33.3)		.046
Ever smoked	37 (48.1)		30 (66.7)		
Genetic characteristics					
*APOE*, No. (%)					
Ɛ4^+^	22 (30.6)		11 (26.2)		.62
Ɛ4^−^	50 (69.4)		31 (73.8)		
Metabolic characteristics, Mean (SD)					
Glucose, mmol/L	77	5.4 (0.5)	45	6.0 (0.8)	<.001
Triglycerides, mmol/L	77	1.1 (0.4)	45	2.0 (0.8)	<.001
Cholesterol, mmol/L	77	5.0 (0.8)	45	5.1 (1.1)	.94
HDL, mmol/L	77	1.5 (0.3)	45	1.3 (0.3)	.01
LDL, mmol/L	77	3.0 (0.7)	45	2.9 (1.0)	.99
Total cholesterol/HDL	77	3.5 (0.9)	45	4.3 (1.3)	<.001
Insulin, pmol/L	76	46.7 (30.5)	45	73.4 (34.1)	<.001
HOMA	76	1.6 (1.2)	45	2.9 (1.8)	<.001
hsCRP, mG/L	76	1.2 (1.6)	44	2.3 (3.4)	.039
Blood pressure, Mean (SD), mmHg					
Systolic BP	77	123.0 (12.4)	45	133.0 (16.4)	.002
Diastolic BP	77	73.6 (7.3)	45	77.1 (7.2)	.10
MAP	77	89.9 (8.3)	45	95.6 (9.2)	.007
Metabolic syndrome factors, No. (%)					
High waist circumference	20 (26.0)		33 (73.3)		<.001
High triglycerides	4 (5.2)		31 (68.9)		<.001
Low HDL	9 (11.9)		25 (55.6)		<.001
Hypertensive	29 (37.7)		39 (86.7)		<.001
High glucose	28 (36.4)		37 (82.2)		<.001
Medications, No. (%)					
Antihypertensive	14 (18.2)		24 (53.3)		<.001
Antihyperglycemic^b^	1 (1.3)		2 (4.4)		.26
Antihyperlipidemic	7 (9.1)		14 (31.1)		.002

MetS, metabolic syndrome; BMI, Body mass index; $\dot{V}O_{2\max}$, Maximal oxygen uptake; *APOE*, Apolipoprotein E; HDL, high‐density lipoprotein cholesterol; LDL, Low‐density lipoprotein cholesterol; HOMA, Homeostatic model assessment; HsCRP, High‐sensitivity C‐reactive protein; BP, Blood Pressure; MAP, Mean arterial blood pressure.

a Calculated from *t* test or *χ* ^*2*^

b Probability calculated from *fisher exact* test.

**Table 4:** Table of collinearity diagnostics among study variables

Collinearity diagnostics										
Variable	Eigen value	Condition index	Proportion of Variation							
			Intercept	MetS	Age	Sex	Education	Smoking status	Drinking status	*APOE ε*4
Intercept	4.3854	1	0.01	0.02	0.01	0.02	0.01	0.01	0.01	0.01
MetS	0.9263	2.1759	0.00	0.09	0.01	0.00	0.02	0.01	0.56	0.11
Age	0.7748	2.3791	0.00	0.03	0.02	0.00	0.00	0.09	0.24	0.49
Sex	0.5827	2.7434	0.00	0.52	0.08	0.10	0.03	0.02	0.18	0.06
Education	0.4767	3.033	0.00	0.31	0.13	0.43	0.02	0.16	0.00	0.00
Smoking status	0.4053	3.2895	0.00	0.03	0.42	0.29	0.04	0.32	0.00	0.05
Drinking status	0.3534	3.523	0.02	0.00	0.24	0.08	0.24	0.30	0.01	0.25
*APOE ε*4	0.0954	6.7796	0.96	0.01	0.07	0.08	0.64	0.08	0.00	0.03

MetS, metabolic syndrome; *APOE*, Apolipoprotein E.

The association between MetS adjusted for independent factors (age, sex, education, smoking history, drinking status, and *APOE* genotype) and the cerebrovascular health indices is shown in Table 5. In the models, these independent factors explained between 5.8% and 28.6% of the total variation in the cerebrovascular indices. Overall, after adjusting for age, sex, education, smoking history, drinking status, and *APOE* genotype, MetS predicted poorer cerebrovascular health as indicated by lower CVC, ${\bar{V}}_{P}$, CVC‐reactivity, and ${\bar{V}}_{P}$‐reactivity (Table 5).

**Table 5:** Linear regression model between outcomes of cerebrovascular health and the different predictors

Outcome variable	Predictor	*β* Regr Coeff (SE)	*P*‐value	Model *R*² (%)
CVC	Intercept	0.6695 (0.0278)***	<.001	28.61***
	Age	−0.0662 (0.0204)**	0.001	
	Sex	−0.1305 (0.0213)***	<.001	
	MetS	−0.0814 (0.0234)***	<.001	
	Education	0.0164 (0.0236)	0.49	
	Smoking status	0.0372 (0.0212)	0.082	
	Drinking status	0.0675 (0.0364)	0.065	
	*APOE ε*4	0.0393 (0.0231)	0.091	
${\bar{V}}_{P}$	Intercept	57.7028 (2.1273)***	<.001	22.42***
	Age	−6.0853 (1.5635)***	<.001	
	Sex	−7.9248 (1.6333)***	<.001	
	MetS	−4.2978 (1.7902)*	0.017	
	Education	1.2278 (1.8093)	0.498	
	Smoking status	2.6996 (1.6259)	0.098	
	Drinking status	3.1331 (2.7878)	0.262	
	*APOE ε*4	2.4983 (1.7679)	0.159	
CVC‐reactivity	Intercept	0.0156 (0.0017)***	<.001	5.8
	Age	−0.0017 (0.0012)	0.168	
	Sex	−0.0017 (0.0013)	0.189	
	MetS	−0.0034 (0.0014)*	0.02	
	Education	0.0002 (0.0014)	0.867	
	Smoking status	0.0003 (0.0013)	0.812	
	Drinking status	0.0023 (0.0022)	0.312	
	*APOE ε*4	0.0005 (0.0014)	0.728	
${\bar{V}}_{P}$‐reactivity	Intercept	2.2936 (0.1704)***	<.001	10.32**
	Age	−0.2062 (0.1253)	0.101	
	Sex	−0.2364 (0.1309)	0.072	
	MetS	−0.3279 (0.1434)*	0.023	
	Education	0.0946 (0.1450)	0.515	
	Smoking status	0.0739 (0.1303)	0.571	
	Drinking status	0.5154 (0.2234)*	0.022	
	*APOE ε*4	0.2991 (0.1416)*	0.036	

${\bar{V}}_{P}$, cerebral blood flow; CVC, cerebrovascular conductance; ${\bar{V}}_{P}$‐reactivity, cerebral blood flow reactivity to hypercapnic challenge from +1 torr to +8 torr; CVC‐Reactivity, cerebrovascular conductance reactivity to a hypercapnic challenge from +1 torr to +8 torr; MetS, metabolic syndrome; *APOE*, Apolipoprotein E.

***P < 0.001, **P < 0.01, *P < 0.05.

Female participants had higher CVC and ${\bar{V}}_{P}$ than male participants. There were no differences in CVC‐reactivity and ${\bar{V}}_{P}$‐reactivity values between male and female participants. Participants with the *APOE ε*4^+^ genotype had higher ${\bar{V}}_{P}$‐reactivity (*P *=* *0.04). There were no differences in the CVC, ${\bar{V}}_{P}$, and CVC‐reactivity levels for *APOE ε*4^+^ participants and *APOE ε*4^−^ participants after adjusting for sociodemographic and lifestyle factors and the presence of MetS. These differences in cerebrovascular outcomes are observed according to the predictor variables, but there was no evidence of an effect of modification (i.e., interaction) of the variables.

## Discussion

This study investigated the effects of MetS on indices of cerebrovascular health in a volunteer sample of community dwelling middle‐aged and older adults. Participants with MetS had lower CVC, ${\bar{V}}_{P}$, CVC‐reactivity, and ${\bar{V}}_{P}$‐reactivity after adjusting for sociodemographic and lifestyle factors and *APOE ε*4 genotype. These results indicate that participants classified with MetS were at higher risk of developing cerebrovascular impairments (Yates et al. 2012). Conversely, *APOE ε*4 genotype was a significant predictor of increased ${\bar{V}}_{P}$‐reactivity and a nearly statistically significant (*P *=* *0.091) predictor for CVC in adjusted analysis. These results suggest that increased vascular reactivity and potentially increased conductance, could be cerebrovascular compensation mechanisms for the deleterious effects of *APOE ε*4 on the brain (Luckhaus et al. 2008; Dai et al. 2009; Wierenga et al. 2012). Given a sample size of 258 subjects and 7 predictors of cerebrovascular indices, these multiple linear regression models are adequately powered to detect statistical significance between each index and predictor. More specifically, this study had more than 90% power to detect a statistical significant association between each vascular index and the MetS status in our models, where *R* ^2^ ranged from 5.8% to 28.6%. These novel and statistically robust findings in a sample of healthy, cognitively intact, middle‐aged, and older adults provide new understanding of the relationship between MetS, *APOE* genotype, and cerebrovascular health indices.

Using magnetic resonance imaging arterial spin labeling Birdsill et al. (2013) found a 15% reduction in mean gray matter CBF in older adults (mean age = 60.4 years) with MetS. With the inclusion of *APOE* genotype, our results are consistent with these findings as we demonstrated a 9.3% reduction in ${\bar{V}}_{P}$ (as assessed by transcranial Doppler ultrasound), equivalent to more than a decade of cerebrovascular aging (Grolimund and Seiler 1988). In addition, we calculated the effect sizes of TCD ${\bar{V}}_{P}$ and ${\bar{V}}_{P}$‐reactivity from our sample (BIM) and from the literature (Giannopoulos et al. 2010; Birdsill et al. 2013) (Table 6). The mean differences between MetS and No MetS in vascular measures in our study were similar to what have been previously reported in which the mean differences for ${\bar{V}}_{P}$ were significant, while the mean differences for ${\bar{V}}_{P}$‐reactivity were not.

**Table 6:** Effect size: Mean difference between MetS and No MetS among the cerebrovascular indices

Cerebrovascular outcome	Study	MetS	No MetS	Mean difference [95% CI]
TCD ${\bar{V}}_{P}$	BIM	49.51	54.57	5.06 [1.77;8.36]
TCD ${\bar{V}}_{P}$	Birdsill et al. 2013	45.90	52.98	7.08[6.74;7.42]
TCD ${\bar{V}}_{P}$‐Reactivity	BIM	2.02	2.26	0.236 [−0.006;0.479]
TCD ${\bar{V}}_{P}$‐Reactivity	Giannopoulos et al. 2010	2.27	2.68	0.41[−0.15;0.97]

${\bar{V}}_{P}$, cerebral blood flow; CVC, cerebrovascular conductance; ${\bar{V}}_{P}$‐reactivity, cerebral blood flow reactivity to hypercapnic challenge from +1 torr to +8 torr; CVC‐reactivity, cerebrovascular conductance reactivity to a hypercapnic challenge from +1 torr to +8 torr; MetS, metabolic syndrome; 95% CI, 95% Confidence Interval.

In our study of middle‐aged and older adults, there was a higher MetS prevalence among *APOE ε*4 carriers than noncarriers. This is consistent with previous research (Olivieri et al. 2007). APOE plays a role in lipid metabolism as it serves as a transport molecule of triglyceride‐rich particles and high‐density lipoprotein cholesterol (Sima et al. 2007). The polymorphisms of the *APOE* gene reflect differing levels of APOE expression with the *ε*4 isoform being expressed the least and is therefore the least efficient than the other isoforms (Sima et al. 2007). Since MetS is associated with an imbalance in lipid and lipoprotein metabolism, the role of the *APOE* genotype may explain results from previous studies that have found impairments in cerebrovascular reactivity to hypercapnia in atherosclerotic subjects with MetS (Giannopoulos et al. 2010). Other mechanisms underpinning the relationship between MetS and *APOE* genotype on cerebrovascular function need to be elucidated, but could include insulin resistance, hyperinsulinemia, inflammation, oxidative stress, and/or endothelial dysfunction (Davenport et al. 2012). Our results extend previous literature showing that hyperinsulinemia and insulin resistance (as indicated by HOMA) are correlated with lower ${\bar{V}}_{P}$ and CVC (Giannopoulos et al. 2010; Yates et al. 2012). These results are supported by the growing literature suggesting that glucose intolerance are linked to higher incidence of stroke (Air and Kissela 2007) and cognitive impairment (Yaffe et al. 2014).

The effects of MetS on brain integrity are arguably partly dependent on the cerebrovascular reactivity impairments (Giannopoulos et al. 2010; Novak et al. 2011; Farooqui et al. 2012). Cerebrovascular reactivity is necessary to deliver oxygen and glucose and to maintain energy‐dependent processes such as regional brain activation by clearing metabolic waste produced by neuronal activity (e.g., CO_2_, other metabolites, heat) (Davenport et al. 2012). Patients with MetS have impairments in endothelial‐dependent vasodilatation (Kaur 2014). Consequently, these individuals may not be able to maintain an optimal neuronal environment (i.e., brain blood flow), particularly during periods of high demand or at advanced ages (Yates et al. 2012). While it is possible that reductions in cerebrovascular reactivity may be associated with direct or indirect deleterious effects of hyperinsulinemia, insulin resistance, and associated inflammation on the microvasculature (Giannopoulos et al. 2010; Kaur 2014), we did not find that hyperinsulinemia, insulin resistance (HOMA), and hsCRP were significant predictors of cerebrovascular reactivity. Clinically, impaired cerebrovascular reactivity predicts an increased risk for ischemic strokes (Silvestrini et al. 2000) and cardiovascular, and all‐cause mortality (Portegies et al. 2014).

In our study, we observed that after controlling for demographic and behavioral factors, *APOE ε*4 carriers had increased ${\bar{V}}_{P}$‐reactivity. These results are consistent with previous studies in older adults in which *APOE ε*4 carriers had increased CBF in specific brain regions such as the hippocampus and precuneus (Wierenga et al. 2013; Zlatar et al. 2014). These and other posterior brain regions are affected early in the onset of AD and are often compromised prior to cognitive deficits (Wierenga et al. 2012). While the *APOE ε*4 allele accounts for approximately 40% of late‐onset AD diagnoses (Devanand et al. 2005; Wierenga et al. 2012), it has been proposed that the increase in CBF observed in older adults with the *APOE ε*4 allele (Wierenga et al. 2012, 2013; Zlatar et al. 2014), and in patients with MCI (Dai et al. 2009) or mild AD, relate to functional compensatory mechanisms to protect the potentially compromised brain (Luckhaus et al. 2008). It has been hypothesized that during the transition to clinically evident cognitive changes (i.e., MCI, dementia) there is a compensatory response within the brain as in response to pathologic vascular changes (Luckhaus et al. 2008; Dai et al. 2009; Wierenga et al. 2012). Future examination of the relationship between MetS and *APOE* genotype will be warranted to investigate how MetS contributes to the observed increase in ${\bar{V}}_{P}$‐reactivity in carriers of *APOE ε*4, but not ${\bar{V}}_{P}$, CVC, or CVC‐reactivity.

The use of ${\bar{V}}_{P}$ for TCD studies as surrogate for cerebral blood flow has been validated previously in vitro and in vivo studies (Arts and Roevros 1972; Bishop et al. 1986; Aaslid et al. 1989; Poulin and Robbins 1996; Poulin et al. 1996; Hatab et al. 1997; Serrador et al. 2000). Still, it is important to discuss some limitations of the technique. ${\bar{V}}_{P}$ is described as the velocity associated with the maximal frequency of the Doppler shift and is the most commonly reported index in TCD studies (Poulin et al. 1996, 1998). This measure assumes that the axial flow velocity is proportional to laminar flow if the angle of insonation is near zero. The application of TCD studies to intracranial vessels such as the MCA is such that this condition is almost always met. In addition, the use of ${\bar{V}}_{P}$ in TCD studies also assumes that the diameter of the MCA remains constant. To circumvent this issue, previous studies have used a validated flow index (Arts and Roevros 1972), which is the product of the intensity weighted mean velocity (${\bar{V}}_{\mathrm{IWM}}$) and the total reflected power signal (Poulin and Robbins 1996; Poulin et al. 1996). Arts and Roevros (1972) elegantly derive from first principles the relationship between the average velocity across the cross‐section of a blood vessel and the power density spectrum of the received signal. They go on to show how the Doppler signal power is proportional to the cross‐sectional area of the vessel. Thus, it is possible to use the indices derived by Arts and Roevros (1972) with transcranial Doppler ultrasound to account for changes that occur in the cross‐sectional area of the vessel being insonated. Indeed, during moderate euoxic hypercapnia levels, previous studies (Poulin and Robbins 1996; Poulin et al. 1996) using an approach similar to the one used in this study have demonstrated that there is minimal change in the cross‐sectional area of the MCA.

Two recent magnetic resonance imaging studies have challenged the relationship between manipulations in $P_{{\mathrm{ETCO}}_{2}}$ and diameter of the middle cerebral artery (Coverdale et al. 2014; Verbree et al. 2014). Coverdale et al. (2014) reported that the middle cerebral artery cross‐sectional area increases by 16% during a hypercapnic challenge achieved by having participants breathe a fixed inspired concentration (i.e., 6%) FiCO_2_. This fixed inspired concentration of CO_2_ resulted in approximately a 10 mmHg (1.2 kPa) increase in $P_{{\mathrm{ETCO}}_{2}}$. The exact physiological stimulus (i.e., the increase in arterial $P_{{\mathrm{CO}}_{2}}$) is very likely to have been different in all their participants because the protocol did not control the actual end‐tidal (i.e., arterial) $P_{{\mathrm{CO}}_{2}}$, and it is known that there is significant interindividual variability in the ventilatory and cerebrovascular responses to alterations in arterial $P_{{\mathrm{CO}}_{2}}$. Thus, this is an important technical consideration concerning the Coverdale study. In our study, we controlled the end‐tidal (i.e., arterial) $P_{{\mathrm{CO}}_{2}}$ at three different steady‐state levels (+1, +5, +8 mmHg above resting end‐tidal $P_{{\mathrm{CO}}_{2}}$ levels), resulting in the same stimulus administered to each of the 258 subjects in our study.

In contrast to the Coverdale paper (2014), Verbree et al. (2014) did not find a change in the diameter of the middle cerebral artery in response to an increase in $P_{{\mathrm{ETCO}}_{2}}$, which was similar to that used in our study (+1 kPa or 7.5 mmHg). Furthermore, Verbree et al. (2014) also controlled $P_{{\mathrm{ETCO}}_{2}}$ levels relative to participants’ baseline (i.e., resting) values. The findings by Verbree et al. (2014) corroborate earlier findings by Serrador and colleagues (2000) in which $P_{{\mathrm{ETCO}}_{2}}$ was increased in the lower range and did not observe an increase in the cross‐sectional area of the middle cerebral artery. The reason(s) for discrepancies between MRI studies examining the relationship between CO_2_ administration and middle cerebral artery diameter are not clear, but are likely to be due to factors such as different MRI approaches (3 Tesla in Coverdale study vs. 7 Tesla in Verbree study) and/or inherent assumptions with each analytical approach, as well as differences in hypercapnia protocols.

Finally, the study by Verbree and colleagues corroborates the work by Serrador et al. (2000), who have been widely cited as validation of the use of TCD (using cerebral blood flow velocity) as a surrogate measurement of cerebral blood flow when examining vascular reactivity during hypercapnia at the lower limits (i.e., up to +8 mmHg). Moreover, a recent study by Spencer et al. (2015) assessed the stability of resting cerebral blood flow and cerebrovascular reactivity over 6 months in a similar cohort of older adults as the one used in this study. They demonstrated that peak middle cerebral artery velocity and reactivity were stable over a 6‐month period when normalized to $P_{{\mathrm{ETCO}}_{2}}$. These findings demonstrate the importance of considering MCA velocity data in the context of $P_{{\mathrm{ETCO}}_{2}}$ levels. Collectively from these studies, it would appear that at the lower levels of hypercapnia, there is no significant detectable change in the diameter of the middle cerebral artery. Thus, the use of TCD as a surrogate measure for cerebral blood flow seems appropriate for studies of cerebrovascular regulation, under controlled conditions such as those outlined in our study and others (Serrador et al. 2000; Verbree et al. 2014; Spencer et al. 2015).

A limitation of this study is that we did not assess the impact of medication use on the associations between cerebrovascular health markers and MetS. In addition, as a cross‐sectional study, a causal relationship cannot be determined. Future research investigating the effects of exercise and/or nutritional interventions known to modulate MetS (Donley et al. 2014; Kaur 2014) is warranted to better understand the mechanisms by which MetS affects cerebrovascular health. Contrary to prior studies we found that education, smoking history, and drinking status were not statistically significant predictors of cerebrovascular health indices (Slagter et al. 2014). These findings may be explained by the fact that our study sample showed little variation on some of these factors. For example, 89% of the participants are well educated, 54% have never smoked, and 91% were light drinkers. Participants in this report were all considered “healthy” middle‐aged and older adults with none exhibiting clinically overt vascular disease. However, despite this, similar to reported North American MetS prevalence (Ervin 2009; Statistics Canada, 2014), about 30% of our sample was still classified with MetS.

In summary, MetS is an important risk factor associated with cerebrovascular health indices in our study of community‐dwelling older adults. Carriers of *APOE ε*4 had higher CVC and ${\bar{V}}_{P}$‐reactivity. With the expected rise in MetS prevalence, these results support the need for future investigations examining the impact of interventions such as exercise (Kemmler et al. 2013) and/or nutritional modifications (Brader et al. 2014) on reducing both the development and the consequences of the MetS and its effects on the cerebral vasculature.

## Conflict of Interest

None declared.
